# Seasonal dynamics of DNA and RNA viral bioaerosol communities in a daycare center

**DOI:** 10.1186/s40168-019-0672-z

**Published:** 2019-04-01

**Authors:** Aaron J. Prussin, Pedro J. Torres, John Shimashita, Steven R. Head, Kyle J. Bibby, Scott T. Kelley, Linsey C. Marr

**Affiliations:** 1 Department of Civil and Environmental Engineering, Virginia Tech, Blacksburg, VA 24061 USA; 2 Department of Biology, San Diego State University, San Diego, CA 92182 USA; 3 Next Generation Sequencing and Microarray Core Facility, The Scripps Research Institute, La Jolla, CA 92037 USA; 4 Department of Civil and Environmental Engineering, University of Notre Dame, Notre Dame, IN 46556 USA

**Keywords:** Virome, Metagenomics, Built environment, Bioaerosol

## Abstract

**Background:**

Viruses play an important role in ecosystems, including the built environment (BE). While numerous studies have characterized bacterial and fungal microbiomes in the BE, few have focused on the viral microbiome (virome). Longitudinal microbiome studies provide insight into the stability and dynamics of microbial communities; however, few such studies exist for the microbiome of the BE, and most have focused on bacteria. Here, we present a longitudinal, metagenomic-based analysis of the airborne DNA and RNA virome of a children’s daycare center. Specifically, we investigate how the airborne virome varies as a function of season and human occupancy, and we identify possible sources of the viruses and their hosts, mainly humans, animals, plants, and insects.

**Results:**

Season strongly influenced the airborne viral community composition, and a single sample collected when the daycare center was unoccupied suggested that occupancy also influenced the community. The pattern of influence differed between DNA and RNA viromes. Human-associated viruses were much more diverse and dominant in the winter, while the summertime virome contained a high relative proportion and diversity of plant-associated viruses.

**Conclusions:**

This airborne microbiome in this building exhibited seasonality in its viral community but not its bacterial community. Human occupancy influenced both types of communities. By adding new data about the viral microbiome to complement burgeoning information about the bacterial and fungal microbiomes, this study contributes to a more complete understanding of the airborne microbiome.

**Electronic supplementary material:**

The online version of this article (10.1186/s40168-019-0672-z) contains supplementary material, which is available to authorized users.

## Background

There is an emerging emphasis on understanding the complex interactions among humans, the built environment (BE), and the microbiomes of both because of their effects on human health [1]. This thrust has been facilitated by applications for molecular methods that have dramatically expanded our understanding of the diversity and dynamism of microbes [2–4]. While numerous studies have characterized bacterial and fungal microbiomes in the BE [2, 5–9], far fewer have focused on the viral microbiome (virome). Williams [10] described viruses as “the forgotten siblings of the microbiome family” and argued that the human virome is probably at least as important for human health as is the “bacteriome.” Viruses play critical roles in many ecosystems. For instance, many bacteriophages act as predators to control bacterial populations [11–14], while others determine bacterial pathogenicity by directly inserting pathogenicity islands and virulence factors into bacterial genomes [15]. Numerous eukaryotic viruses impact human health, and some can spread rapidly through the BE as airborne particles, known as “aerosols” [16–20]. We have shown previously that viruses in indoor air are as numerous as bacteria [21].

One reason for the paucity of data on the airborne virome is the inherent technical difficulty of studying it [22]. Some of the challenges of studying airborne virome include no conserved viral gene for marker studies (e.g., 16S for bacteria and ITS/18S for fungi), sampling challenges particularly for aerosols, low amounts of biomass in the air, limited representation of viruses in reference databases, and the often short-term persistence of viruses in the environment, especially RNA viruses [22]. While several studies have investigated the spread of pathogenic viruses (e.g., norovirus and rotavirus) on surfaces in the BE [23–25], few have attempted to apply broad-scale deep-sequencing approaches to analyzing entire viromes in the BE [26].

As a result, studies on the virome of the BE and/or the airborne virome have been sparse. One study that examined the DNA virome on restroom surfaces reported that enterophages, human papilloma virus, and herpesviruses were the most abundant viruses [26]. We are aware of five studies that have characterized the airborne virome [27–31]. Three of these (Hall et al. [28], Brisebois et al. [30], and Rosario et al. [31]) included samples from the BE, and none examined longitudinal effects. Longitudinal data are important because they can provide insight into the stability and dynamics of microbial communities. Longitudinal studies can also reveal temporal or seasonal trends while assisting with causal inference (e.g., viruses associated with certain seasonal diseases are more prevalent in the winter).

Results of studies on the bacterial and fungal communities indoors offer clues but not a complete picture about the dynamics of the airborne virome. Seasonal variations in the bacterial community in outdoor air [32] can theoretically propagate to indoor air, as bioaerosols are readily exchanged between the two volumes [33]. A study in Finland observed seasonal patterns of bacterial diversity in indoor dust [34], while one in the San Francisco Bay Area found only weak evidence of seasonality in bacterial diversity [35]. In both cases, differences were greater between buildings or even between rooms within the same building than between seasons, indicating that building characteristics and/or occupants are more important than season in shaping the bacterial microbiome. In terms of BE fungal communities, Adams et al. [35] found that fungal diversity in settled dust was strongly influenced by season and appeared to be dominated by outdoor sources. These studies have shown that a combination of outdoor air, occupants, and building characteristics contributes to shaping the microbiome of the BE [2, 36].

The seasonality of human-associated viruses, such as influenza virus and rotavirus [37–39], suggests that there may also be seasonal patterns in at least part of the virome in the BE. Viruses that infect bacteria, known as bacteriophage or phage, are primary drivers of the structure and function of bacterial communities in many environments [13, 14, 40]. Given that seasonal trends have been observed in the bacterial microbiome in some BEs, it is plausible their phage predators exhibit parallel seasonal dynamics.

Because of the long hours that some children spend at daycare centers, these buildings are of special interest in understanding linkages between the microbiome of the BE and human health. Furthermore, daycare centers could potentially harbor a large diversity and number of viruses, thanks in part to high occupancy by young children who have yet to develop hygienic behaviors [41, 42]. Daycare centers also represent an ideal test environment to understand the spread of viral diseases in the classrooms of schools or universities. Thus, daycare centers are an excellent model system in which to study both the diversity of airborne viruses and their possible seasonality. In our prior study of the seasonal dynamics of airborne bacteria in a daycare center, we found no significant seasonal differences in community structure by season [43]. Herein, we analyze samples that were collected continuously over the course of a year in a daycare center to test the hypothesis that the airborne viral microbiome is influenced by both seasonality and human occupancy. We extracted DNA and RNA from the samples and performed deep-sequencing and a metagenomics analysis. We found that both season and human occupancy had a strong influence on the DNA and RNA viral community composition, as well as specific viral taxa. Furthermore, human-associated viruses were more abundant in the winter while plant-associated viruses were more abundant in the summer. These results complement knowledge about airborne bacterial and fungal microbiomes and contribute to a more complete understanding of the airborne microbiome in the BE.

## Results

### Most abundant viruses in a daycare center

To characterize the airborne viral community in a daycare center, we collected filter samples from the heating, ventilation, and air conditioning (HVAC) system between January 2014 and February 2015, as previously described [43]. We analyzed a total of three ~ 1-month-long samples from each of spring, summer, and fall and four samples from winter. Additionally, we collected a sample when the daycare center was closed in late December and early January. The daycare center only closes for an extended period of time once per year, and thus, we were only able to collect one sample while it was closed in this study. We also analyzed two negative controls (unexposed filter and molecular biology grade water, for DNA and RNA samples), which showed an extremely low total BLAST hit output of DNA (mean = 100) and RNA (mean = 2) viral hits (Additional file 1: Figure S1 a-b) compared to the daycare samples (DNA mean = 3892 and RNA mean = 2320).

**Fig. 1:**
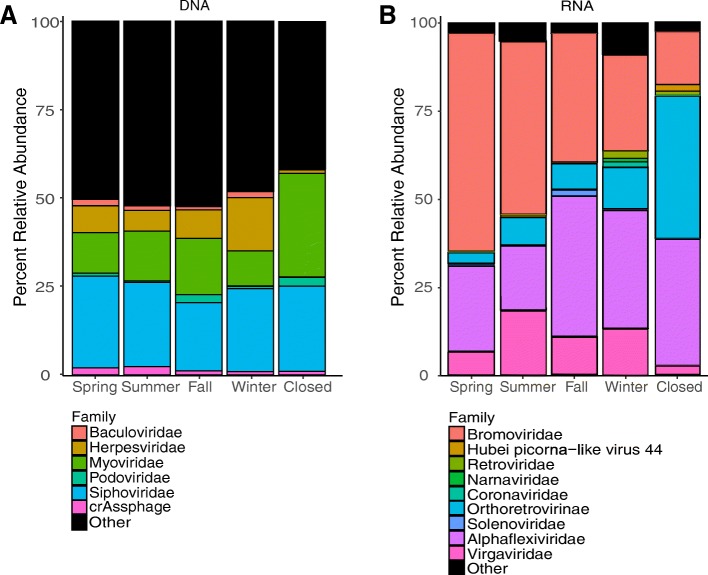
Mean relative abundance of airborne DNA and RNA viruses at the level of family by season. Stacked barplot showing the percent relative abundance of the top 20 most abundant **a** DNA and **b** RNA airborne viruses at the family level, when possible, summarized by season (spring, *n* = 3; summer, *n* = 3; fall, *n* = 3; winter, *n* = 4; closed *n* = 1)

According to the mean relative abundance of all viruses, the top 20 most abundant DNA viruses belonged to six different taxonomic families (summarized to family level when possible) (Fig. 1a). One of the six viral families was Herpesviridae, a human-associated virus that made up 7.8% of the viruses in the spring, 6.1% in the summer, 8.3% in the fall, 15.4% in the winter, and 1.2% when the facility was closed. An arthropod-associated virus, Baculoviridae, comprised 2.1% of the viral sequences in the spring, 1.6% in the summer, 1.1% in the fall, 1.9% in the winter, and 0.6% when the daycare center was closed. Four out of six viral families were bacteriophages, including Myoviridae, Podoviridae, Siphoviridae, and crAssphage. Together, these bacteriophage families represented 42% of the identified viral sequences in the spring, 43% in the summer, 41% in the fall, 37% in the winter, and 59% when the daycare center was closed.

According to the mean relative abundance of all viruses, the top 20 most abundant RNA viruses belonged to nine different families (summarized to family level when possible) (Fig. 1b). Four out of nine viral families were plant-associated: Bromoviridae, Solemoviridae, Alphaflexiviridae, and Virgaviridae. Together, these viruses comprised a mean of 94% of the total RNA viruses in the spring, 86% in the summer, 89% in the fall, 74% in the winter, and 54% when the daycare center was closed. One belonged to the family Retroviridae which was largely composed of the human endogenous retrovirus, which represented 0.33% of the total in the spring, 0.65% in the summer, 0.22% in the fall, 2.1% in the winter, and 1.2% when the daycare center was closed. The Hubei-picorna-like virus (arthropod host) represented < 0.2% of the total throughout all seasons except when the daycare was closed (1.9%). The Orthoretrovirinae family represented 3% of the viruses in the spring, 7.8% in the summer, 7.2% in the fall, 12.1% in the winter, and 40.5% when the daycare center was closed. The Narnaviridae family (fungal host) represented 0.18% of the viruses in the spring, 0.13% in the summer, 0.18% in fall, 1% in the winter, and 0% when the daycare center was closed. Both animals and humans serve as a natural host to the Coronaviridae family, which represented 0% of the viruses in the spring, summer, and fall; 1.14% of the viruses in the winter; and 0.23% when the daycare center was closed.

Although this study did not focus on pathogens, we searched for influenza virus and other common pathogens (e.g., adenovirus, RSV, rotavirus). These accounted for < 0.005% of the total relative abundance in all seasons.

### Alpha diversity

**Fig. 2:**
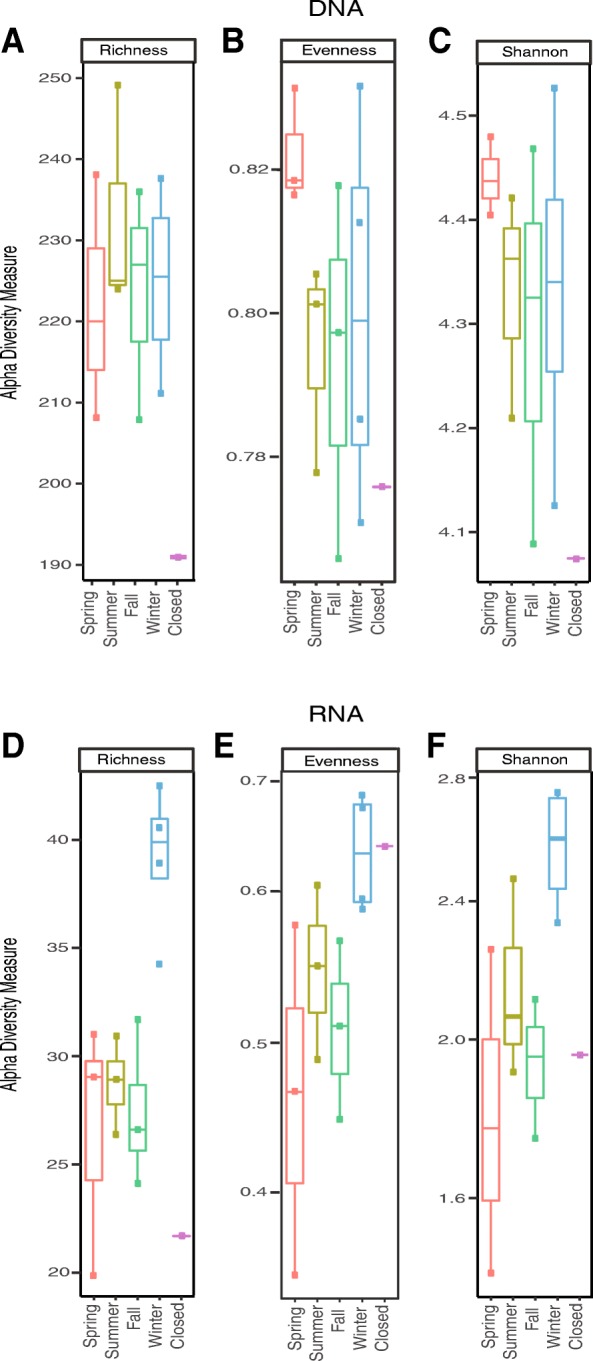
Airborne DNA and RNA viral community richness and evenness by season. Boxplots of alpha diversity by season (spring, *n* = 3; summer, *n* = 3; fall, *n* = 3; winter, *n* = 4; closed *n* = 1); whiskers extend 1.5× past the interquartile range. **a**–**c** Estimates of alpha diversity for DNA viruses based on **a** observed species, **b** Pielou’s evenness, and **c** Shannon diversity index. **d**–**f** Estimates of alpha diversity for RNA viruses based on **d** observed species, **e** Pielou’s evenness, and **f** Shannon diversity index

We investigated how the number of viral species (richness) and their distribution (evenness) in the indoor air virome varied with season and human occupancy by using the number of unique BLAST hits in each sample as an estimate of species abundance for DNA and RNA viruses (Fig. 2). DNA communities tended to have a higher richness as estimated by observed species in the summer (Fig. 2a), while the evenness of the viral community was higher in the spring as measured by the Pielou and Shannon indices (Fig. 2c, d), although this difference was not significant. Notably, the DNA communities exhibited the lowest diversity and evenness when the daycare center was closed (Fig. 2a–c). Seasonality had a more pronounced effect on the diversity of RNA communities. RNA communities tended to have a lower richness and evenness in the spring and higher richness and evenness in the winter (Fig. 2d–f). While not as noticeable as in the DNA communities, there was reduced diversity when the daycare center was closed (Fig. 2d). Unlike the DNA viral communities, RNA viral communities showed very high evenness when the daycare center was closed (Fig. 2e, f).

### Beta diversity

**Fig. 3:**
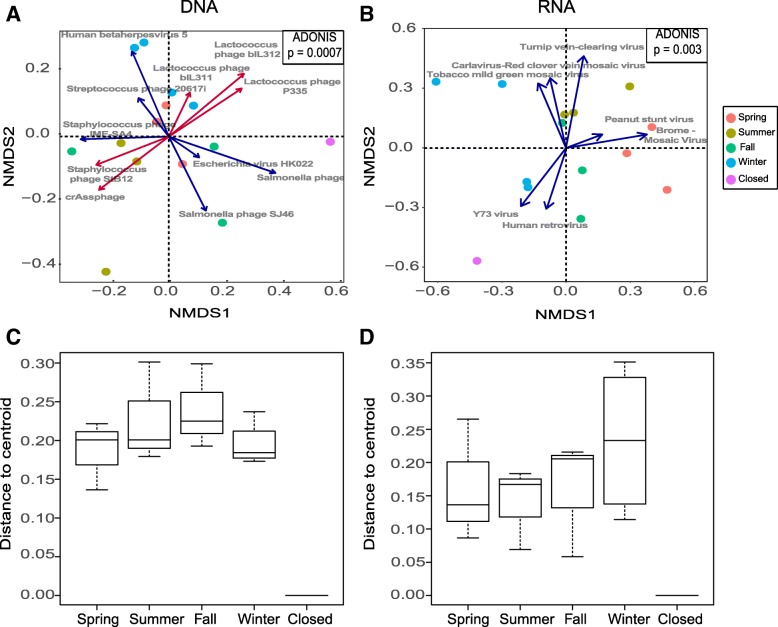
Impact of season and human occupancy on the airborne DNA and RNA viral community structure. **a**, **b** Non-metric multidimensional scaling (NMDS) biplots of **a** DNA and **b** RNA viruses based on Bray-Curtis dissimilarities. Blue arrows indicate positive correlations and red arrows indicate negative correlations between the abundances of specific viruses and the dissimilarity matrix. The arrow length indicates the relative strength of the correlation. Results of Permutation Analysis of Variance (ADONIS function in R vegan package) are shown in the box inset. **c**, **d** Boxplots showing levels of beta dispersion by season and when the center was closed for **c** DNA and **d** RNA viruses, respectively. No statistical differences in the overall beta dispersion for DNA (permutation test, *p* = 0.07) or RNA (permutation test, *p* = 0.46) were detected among seasons

We used non-metric multidimensional scaling (NMDS) plots based on Bray-Curtis dissimilarity to compare the similarity of the airborne DNA and RNA viral community composition between different seasons. There was noticeable clustering between samples from different seasons in the DNA (Fig. 3a) and RNA viral communities (Fig. 3b). Permutation Multivariate Analysis of Variance (PERMANOVA - ADONIS function in R vegan package) showed that season had a significant effect on the viral community structure for both DNA (*p* = 0.0007) and RNA (*p* = 0.003) viruses. We then identified species that best described the overall biological pattern of the dissimilarity matrix and fitted them to the existing NMDS plot, forming a biplot. Arrows were colored to indicate positive correlation (the highest relative abundance in that direction - blue) and negative correlation (the lowest relative abundance in that direction - red). Through our analysis, we found that viral diversity was strongly affected by season. Human-associated viruses were relatively more abundant in the winter (Fig. 3a), while plant pathogenic viruses were relatively more abundant in the spring, summer, and fall (Fig. 3b). Among DNA viruses, Lactococcus phage, Human betaherpesvirus 5 (Cytomegalovirus), and Streptococcus phage increased in relative abundance in the winter; Staphylococcus phage and crAssphage decreased in relative abundance during the fall; and Escherichia virus and Salmonella phage increased in relative abundance in the summer and fall (Fig. 3a). Among RNA viruses, Turnip vein-clearing virus, Brome mosaic virus, and peanut stunt virus increased in relative abundance in the spring and summer (Fig. 3b). There were some viruses whose correlation did not point to a particular cluster such as the Tobacco mild green mosaic virus and Carlavirus-Red clover vein mosaic virus, which seemed to relatively increase in between the winter and summer season (closer to summer). The Y73 virus and Human retrovirus relatively increased around the winter season and when the daycare center was closed. Confidence in differences when the daycare center was closed is limited because we had only one such sample.

We also examined the variability in community structure (assessed by the analysis of beta dispersion) of DNA and RNA viruses within each individual season (Fig. 3c, d). Interestingly, DNA and RNA communities showed different dispersion trends based on the season. For DNA viruses, the variability of the within-season community structure increased from spring to fall and then fell in the winter. For RNA viruses, the variability of the within-season community structure was lowest in the summer and highest in the winter. Though there were modest differences in the beta dispersion, we did not detect statistically significant heterogeneity in the DNA (permutation test, *p* = 0.07) or RNA (permutation test, *p* = 0.28) beta dispersion. This indicates that there is an effect of season on viral communities, and these communities display similar community variance.

### Most important viruses driving seasonality

**Fig. 4:**
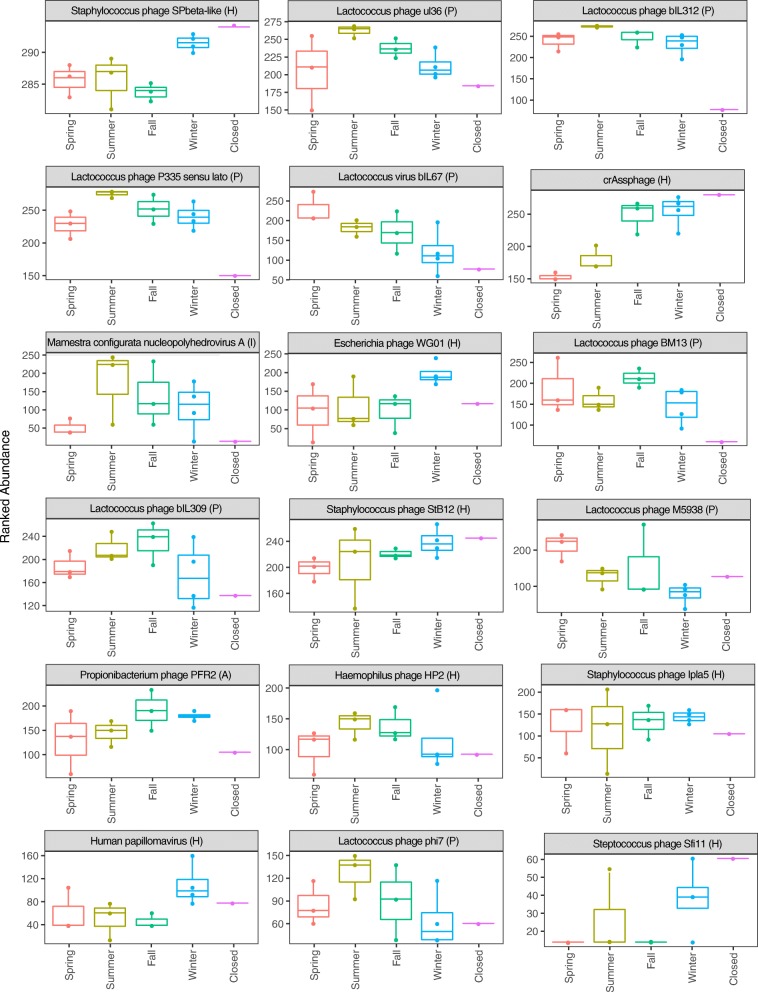
Ranked abundances of DNA viral species that had the strongest seasonality. Random forest modeling was used to identify viruses that best distinguish between the different seasons: spring, summer, fall, and winter and when the daycare center was closed. The ranked abundance of the top 18 most discriminant DNA viruses is shown identified down to the strain level, if possible. *P* = plant-associated virus, *H* = human-associated virus, *A* = animal-associated virus, *I* = insect-associated virus, and S/OP = soil-associated/opportunistic pathogen

**Fig. 5:**
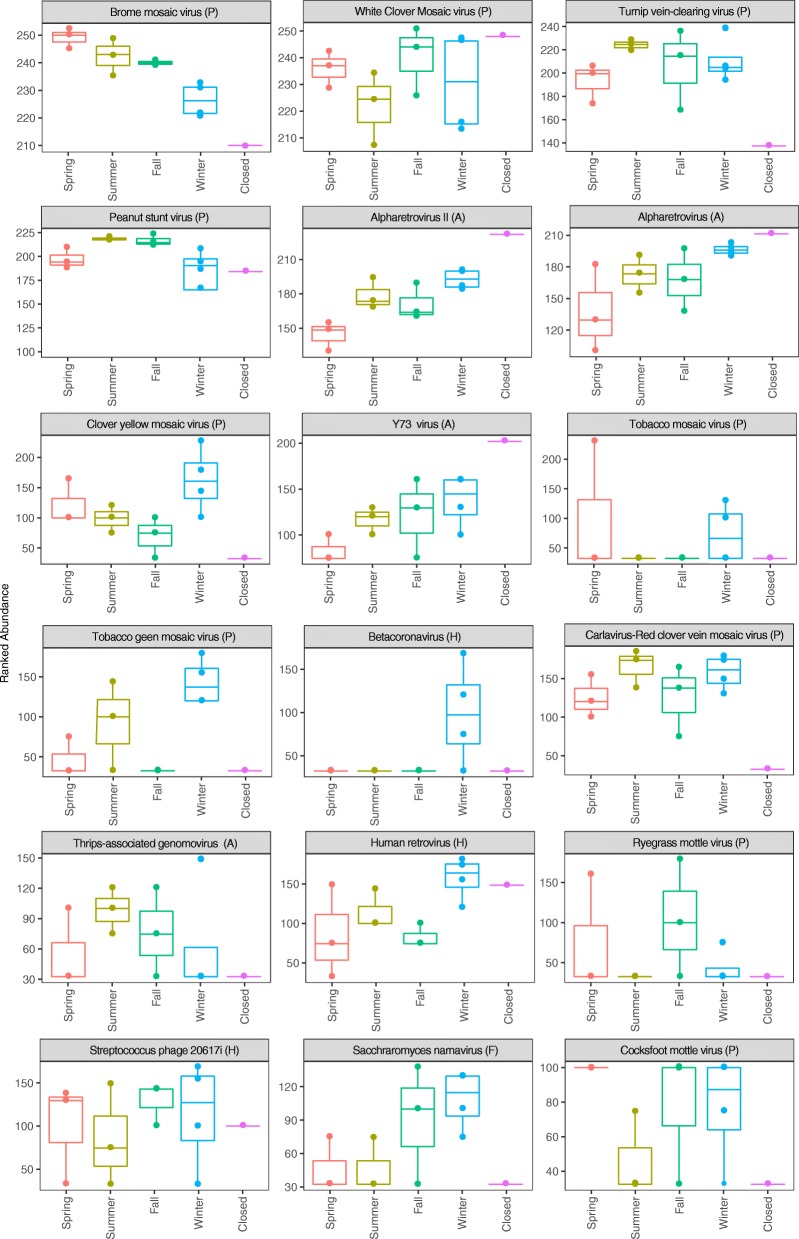
Ranked abundances of RNA viral species that had the strongest seasonality. Random forest modeling was used to identify viruses that best distinguish between the different seasons: spring, summer, fall, and winter and when the daycare center was closed. The ranked abundances of the top 18 most discriminant RNA viruses are shown identified down to the strain level, if possible. *P* = plant-associated virus, *H* = human-associated virus, *A* = animal-associated virus, *I* = insect-associated virus, and *F* = fungal-associated virus

We used a random forest machine learning classifier to determine how well the season could be predicted based on the viral hits. The random forest model predicted the season with 62% accuracy for DNA viruses and 58% for RNA viruses. Variable importance by a mean decrease in accuracy was then calculated for the random forest model. Figure 4 and Fig. 5 illustrate 18 DNA and RNA viruses, respectively, whose removal caused the greatest decrease in model accuracy. Most DNA viruses identified showed clear seasonal trends in the normalized ranked abundance. Out of the 18 viral DNA strains, eight were plant-associated, eight were human-associated, one was animal-associated, and one was insect-associated (respectively denoted by the P, H, A, and I next to the virus name in Fig. 4). Seven of the eight plant-associated viruses showed higher abundances on average in the summer when compared to the winter, while seven out of the eight human-associated viruses had higher numbers on average in the winter when compared to the summer. Interestingly, all viruses that displayed high abundances when the daycare center was closed were human-associated viruses. The *Staphylococcus* phage SPbeta-like, which is associated with human skin, displayed the highest relative abundance of all the viruses, likely due to constant shedding of skin of the human occupants.

RNA viruses also showed clear seasonal trends in their normalized relative abundance. Out of the 18 viral RNA strains, 10 were plant-associated, four were animal-associated, three were human-associated, and one was fungal-associated (respectively denoted by the P, A, H, and F next to the virus name Fig. 5). Five of the 10 plant-associated viruses showed higher abundances in the summer than the winter, and two were higher in the winter (clover yellow mosaic and tobacco green mosaic virus). The three human-associated viruses were more abundant in the winter compared to summer. Except for the thrips-associated genomovirus, which was most abundant in the summer, all animal-associated viruses showed the highest abundance when the daycare center was closed. The RNA viruses consisted of positive sense ssRNA viruses, ssRNA-RT viruses, and dsRNA viruses. All ssRNA-RT viruses (Alpharetrovirus, Y73 virus, and Human retrovirus) were most abundant during the winter and when the daycare center was closed (which was during the winter, Table 1).

**Table 1:** Sampling dates for viral analysis

Sample number	Start date	End date	Season
1	20 January 2014	17 February 2014	Winter
2	17 February 2014	17 March 2014	Winter
3	17 March 2014	14 April 2014	Spring
4	14 April 2014	12 May 2014	Spring
5	12 May 2014	09 June 2014	Spring
6	09 June 2014	07 July 2014	Summer
7	07 July 2014	04 August 2014	Summer
8	04 August 2014	02 September 2014	Summer
9	02 September 2014	29 September 2014	Fall
10	29 September 2014	27 October 2014	Fall
11	27 October 2014	24 November 2014	Fall
12	24 November 2014	23 December 2014	Winter
13	05 January 2015	02 February 2015	Winter
14^a^	23 December 2014	05 January 2015	Closed

^a^Daycare center was closed during this entire period

## Discussion

Both human occupancy and seasonality influenced the airborne virome in the daycare center in this study. When the building was closed and unoccupied, the community structure was significantly different than at all other times of the year, highlighting the apparent role humans play in the viral ecology of this building. A limitation of this study is that we were only able to collect a single sample when the daycare center was unoccupied, as it is closed for an extended period of time only once per year. Future research is needed to confirm the effect of human occupancy through either a multi-year and/or multi-location study.

The importance of human occupancy in shaping the airborne microbiome has been established previously for bacteria [43, 44]; this study shows that humans also shape the airborne virome in the BE. Our results show that human-associated viruses were more abundant in winter, while plant-associated viruses were more abundant in summer in this building. The reason for the higher proportion of human-associated viruses in winter was likely a combination of the children spending a larger fraction of their time indoors (when the weather was warmer, they usually spent at least 2 h per day outside), a lower air-exchange rate due to closed windows, and a higher recirculation rate by the heating system. In contrast, windows were open more often during the warmer months, leading to a higher air-exchange rate and greater influence of viruses from outdoor air, such as plant-associated ones. This pattern could differ in a warmer climate if, for instance, windows remain open more during the cooler months and closed with the air conditioning running during the summer.

There was no significant difference in the richness of DNA viruses between different seasons, and while evenness was higher in the spring, it was not significantly so. Our observation of a lower diversity of DNA viruses when the daycare center was closed is consistent with the idea that humans contribute to the viral richness in the BE. In contrast to DNA viruses, there were seasonal differences in the richness and evenness of RNA viruses; measures of richness and evenness were higher in the winter compared to other seasons (Fig. 2d–f). This observation could be due to the fact that RNA viruses are generally less stable in the environment compared to DNA viruses, and RNA viruses have been shown to persist longer at colder temperatures [45, 46], although this would apply mainly to viruses of outdoor origin, since indoor temperatures do not vary much by season. We also saw a reduction in RNA virus richness when the daycare center was closed, but its evenness was comparable to that of the winter season. When the daycare center was open, there was a higher frequency of low-abundance viruses compared to when the facility was closed. A possible reason for this is that when the daycare center was open, the children and staff contributed a constant influx of viruses from themselves and the outside environment, thereby increasing viral richness. The somewhat counterintuitive increase in evenness during closure appears to result from the fact that there were relatively similar abundances of the persisting viruses.

One of the most striking observations was a noticeable clustering in the virome samples based on the season. The significant effect of seasonality on the virome was notable, as previous work has suggested that although seasonal dynamics help shape the bacterial microbiome in this and other buildings, seasonality is not as significant as other factors (e.g., human occupancy, building location, etc.) [34, 43]. However, for fungi in indoor air, researchers have shown that the community is largely shaped by outdoor air [35, 47]. In sum, our results suggest that a combination of seasonality and human occupancy affects the airborne virome in this daycare center.

A biplot was also used to identify viruses that were the main drivers of seasonal differences in the community. For DNA viruses, we found that the relative abundance of Cytomegalovirus (Human betaherpes virus 5) increased during the winter compared to other seasons, while the relative abundance of Lactococcus phage decreased. Cytomegalovirus infection and transmission occur frequently in daycare centers [48, 49]. This is due to saliva and urine being the primary transmission routes, coupled with the fact that nearly one third of children are infected by age 5 [50]. Lactococci are lactic acid bacteria isolated from numerous sources, including plant surfaces, milk, and animal gastrointestinal tracts [51, 52]. For RNA viruses, the mean relative abundance of plant-associated viruses such as Turnip vein-clearing virus and Carlavirus-Red clover mosaic virus increased in the summer while the abundance of Brome Mosaic Virus and Peanut stunt virus increased in the spring. The blue biplot arrows for the tobacco mild green mosaic virus did not point towards one specific season cluster; however, they did seem to point more towards the summer than the winter season suggesting that their abundance was highest during the summer season. Griffin et al. [53] previously showed that viruses can be transported long distances in the atmosphere, so it is plausible that nearby farms could have been a contributing source. Our results could potentially correlate to the local growing and harvesting season of crops affected by these viruses, although a future source tracking study would be needed to confirm this hypothesis.

Phages were important in driving seasonal variability. Phages with human-associated bacteria as hosts tended to have a higher ranked abundance in the winter compared to the summer. For example, the crAssphage virus, a human gut-associated bacteriophage that is highly abundant in human feces [54], had a higher normalized abundance in the winter than in the summer, consistent with our other results showing greater abundance of human-associated viruses during the winter. A lower air-exchange rate, in combination with frequent changing of diapers, could explain the increased presence of crAssphage in the winter.

The seasonal dynamics in the airborne viral community of daycare center are likely attributable to a combination of human occupancy, building ventilation, and outdoor factors. Human occupancy appears to shape the virome in the wintertime when the building is “tight” and has less air-exchange with outdoors. The large abundance of human skin-associated bacteriophages is not surprising, as humans shed an estimated 14 × 10^6^ bacterial cells per person per day [55]. The increase in abundance of plant-associated viruses in the summer could be explained by an increase in natural ventilation (i.e., windows and doors open more frequently) and possibly their greater abundance in outdoor air. In this geographic region, vegetation is mostly dormant during the winter.

Our results showing clear seasonal dynamics in the indoor air virome contrast with our finding of no seasonality in the bacterial microbiome in this same daycare center [43]. In terms of total number, viruses are approximately equally abundant as bacteria in the air [21], although the seasonal dynamics of numbers and community composition are not known for viruses. The influence of outdoor air on the indoor microbiome could be stronger for viruses than bacteria, as virus-sized particles have a higher penetration efficiency through the building envelope than do larger bacteria-sized particles [56]. Differences in the relative source strength of humans for bacteria vs. viruses could also contribute to differences in seasonal patterns. For fungi, a strong seasonal signal appears to be due to the fact that there are few sources of fungi indoors [47], unless a building suffers from mold. A future source-tracking study, similar to what has been done for bacteria in outdoor air [57], would be valuable for viruses in the BE. Future work is also needed to determine if seasonality also plays an important role in shaping the airborne virome in other buildings and outdoor air.

There are some limitations to this study. Compared to bacteria and fungi, there is a limited representation of viruses in reference databases [22, 58]. Although metagenomics is a very powerful tool to overcome the fact that the vast majority of microbes is not culturable [59], using a sequence-based approach to identify communities does not provide any insight into the viability of the collected viruses. Viability is critical, of course, in understanding these viruses’ role in microbial ecology [60]. Indeed viability is a knowledge gap in many studies examining the microbiome of the BE [61]. Further, all of the numbers reported here are relative abundances, making it impossible to assess whether a higher relative abundance between seasons is due to an increase in certain types of viruses or a decrease in others. Future work would be enhanced by including both viability and absolute abundance in analyses. There is no perfect sample processing protocol for metagenomics, and each step can introduce bias (e.g., filter processing, nucleic acid extraction, sample preparation for sequencing, bioinformatics) [62]. To minimize any potential bias and/or contamination, we included two negative controls (unexposed filter and molecular biology grade water). Both of our controls showed an extremely low number of BLAST hits of DNA and RNA viruses (Additional file 1: Figure S1). RNA degrades more rapidly than DNA, and it is possible some of the RNA degraded before analysis. To minimize any bias and limit degradation, we stored collected samples at − 80 °C, and we analyzed the DNA and RNA viruses independently from each other. Finally, we were able to collect only one sample when the daycare center was closed and unoccupied for an extended period of time. Future studies are needed to examine the effect of occupancy on the airborne virome.

Airborne bacteria and fungi have been relatively well studied in the BE, while viruses have been the “forgotten siblings” [10]. A similar study to that of Kembel et al. [5] examining how building design and operation influences bacteria in the BE should be undertaken for viruses. Researchers should begin to examine how different building parameters (e.g., relative humidity, temperature, and moisture) can affect the virome. For example, relative humidity has been shown to play an important role in the transmission of influenza [63, 64], and it is possible relative humidity could also alter the virome. Additionally, understanding the differences in the airborne virome of the BE between rural and urban settings would address fundamental questions regarding human and plant sources of viruses in the BE.

## Conclusions

Viruses play an important role in human health and the microbial ecology of the built environment, but relatively little is known about the indoor viral microbiome even as we have learned much about the bacterial and fungal communities. We have shown that both human occupancy and season are important in driving the community composition of airborne viruses in a daycare center. Human-associated viruses were much more diverse and dominant in the winter, while the summertime virome contained a high relative proportion and diversity of plant-associated viruses. Armed with a more complete understanding of the airborne microbiome and sources of bioaerosols, biologists, engineers, and architects can work together to optimize the microbiome of the BE for improved health and well-being [5, 65].

## Methods

### Sample collection

We collected filters from the heating, ventilation, and air conditioning (HVAC) system in a daycare center in Blacksburg, VA, USA, between January 2014 and February 2015, with permission from the center’s director and staff as previously described [43]. Previous work has shown that microbial communities collected on HVAC filters were not different from those in air samples collected using an impinger [66]. Blacksburg, Virginia, has a moist continental mid-latitude climate and exhibits four distinct seasons. The center is open from 7:15 am to 5:45 pm Monday through Friday, and a typical day includes organized indoor activities, outdoor play, snack time, lunch time, and nap time. The rooms are cleaned daily, including removal of garbage, vacuuming and mopping of floors, and cleaning of kitchen and bathroom surfaces. The center has a total floor area of 1187 m^2^ (12,800 ft.^2^) split between two buildings, each of which is served by a 4-ton split-system heat pump rated at 2000 ft.^3^ min^−1^ (carrier) [43]. The HVAC system was operating 10–60% of the time when the daycare center was occupied and 38% of the time when the daycare center was closed/unoccupied [43]. Further, the average temperature inside the building ranged between 17 and 21 °C and relative humidity ranged between 26 and 66% throughout the sampling campaign [43]. Every 2 weeks, we removed an exposed filter (Nordic Pure, Tulsa, OK) and installed a new one in an HVAC return duct that was located in a hallway connecting the lobby to the kitchen and four children’s rooms. The filter had a Minimum Efficiency Reporting Value (MERV) rating of 14, meaning that its average particle collection efficiency was > 98%, and its efficiency over the particle size range of 0.3 to 1.0 μm, the most difficult size to collect, was 75–85%. We transported the exposed filter to the laboratory immediately, cut it into ~ 8 cm × 8 cm squares under sterile conditions, placed the filter pieces in a sterile bag (Lansinoh, Alexandria, VA, USA), and froze them at − 80 °C until further processing.

### Sample processing and nucleic acid isolation

To obtain a sample that was representative of about 1 month and maximize the amount of biomass, we combined two 2-week samples into a single, composite sample (Table 1). One square (~ 8 cm × 8 cm) from each filter was cut into smaller pieces (~ 2 cm^2^) and placed into a 50-mL conical tube. We removed the virus particles by vigorously vortexing the filter pieces in ~ 20 mL of 3% beef extract and 0.05 M glycine in molecular biology grade water and then shook them for ~ 15 min at 200 rpm, as previously described [43, 67]. We extracted viral nucleic acid using the QIAamp UltraSens Virus Kit following the manufacturer’s protocol (Qiagen, Calencia, CA, USA). Additionally, we processed two controls (an unexposed filter and a true negative control of molecular biology grade water) identically to the exposed filters. We stored the extracted nucleic acid at − 80 °C until sequencing.

### Sequencing

We fragmented DNA samples by Covaris S2 (intensity setting = 5, duty% = 10, burst cycles = 200, 50 s with frequency sweeping mode). Following the manufacturer recommended protocol, we took the fragmented DNA products into the NEBNext Ultra DNA Library Prep Kit for Illumina with 15 cycles of PCR with no size selection post ligation. We cleaned PCR amplified libraries using 1X AmpureXP Beads and quantified the indexed libraries individually and pooled at equal molar quantity. We gel-purified the pooled libraries by E-Gel EX Agarose Gels, 2% using E-Gel iBase Power System by Thermo Fisher Scientific. Finally, we excised a range of 350–500 bp and sequenced on a NextSeq500 using 2 × 150 paired-end read.

We prepared RNA samples using the Illumnia ScriptSeqv2 RNA-Seq library preparation kit following the manufacturer recommended protocol. The protocol follows a fragmentation at 85 °C for 5 min. Using ScriptSeq index PCR primers, we barcoded the libraries and PCR amplified 15 cycles. We quantified the indexed libraries individually and pooled at equal molar quantity. We gel-purified the pooled libraries by E-Gel EX Agarose Gels, 4% using E-Gel iBase Power System by Thermo Fisher Scientific. Finally, we excised a range of 350–500 bp and sequenced on a NextSeq500 using 2 × 150 paired-end read.

### Sequence analysis

Illumina sequencing yielded an average of ~ 21 million and ~ 8.7 million sequences across all DNA samples and their controls, respectively. An average of ~ 11.3 million and ~ 0.5 million sequences was obtained from RNA samples and their controls, respectively. We trimmed raw paired-end reads using the Trimmomatic (v.0.36) [68] default settings. This was followed by stringent error filtering using PRINSEQ (v.0.20.4) [69] with the following parameters: minimum sequence length of 60 bp, minimum mean quality score of 25, sequences containing any “N’s” were removed and low-complexity threshold of 50 (using Entropy). We used DeconSeq software (coverage > 90, identity > 90) (v.0.4.3) [70] to filter out any human contamination. Following DeconSeq, our paired-end files were rewritten to make sure all reads had a mate and separated out any singletons using FASTQ Pair, available at https://github.com/linsalrob/fastq-pair. We merged overlapping pairs of reads using the default parameters in BBMerge (v.37.36) [71]. We noted that forward reads showed very high-quality scores; therefore, those sequences that did not merge were extracted from the unmerged output file (https://github.com/pjtorres/viral_bioaerosol/xtract_forward.py) as to not discard useful data.

We determined the composition of the metagenome sequence library by using a BLASTn pipeline against a viral RefSeq database. BLASTn (v. 2.6.0) parameters were modified using stringent alignments with *E* value 10^−5^, a sequence identity of 90%, and a minimum raw gapped score of 105. We obtained viral taxonomic lineage using the NCBI accession number for sequences in the reference database and pulling their taxonomic lineage from NCBI (https://github.com/pjtorres/viral_bioaerosol/get_taxlineage_fna.py). We built a custom database containing all viral reference genomes from the NCBI RefSeq database (ftp://ftp.ncbi.nih.gov/refseq/release/viral/). All the scripts used from processing the raw data to BLAST output can be found at https://github.com/pjtorres/viral_bioaerosol. There were 7 DNA viral species and 9 RNA viral species that were removed from further analysis due to their high number of BLAST hits in their respective negative controls. In addition, there were a number of hits to dsDNA and ssDNA viruses in our RNA library. In order to focus solely on RNA viruses we removed viruses whose phylum indicated “dsDNA viruses no RNA stage,” “ssDNA viruses,” and “unclassified bacterial viruses.” In the end, the average total hits in the BLAST output were 3892 for DNA samples and 100 for their controls, and 2302 for the RNA samples and 2 for their negative controls.

We used Rstudio (v.1.0.153) to compute the alpha and beta diversity metrics using phyloseq [72] and vegan [73] packages at a rarefied sampling depth of 1300 for DNA and 850 for RNA viruses. Both of our controls (unexposed filter and molecular biology grade water processed the same as the samples) showed an extremely low relative abundance of DNA and RNA viruses (Additional file 1: Figure S1 a-b). We used three alpha diversity metrics. To estimate species, we used observed species to define the total number of unique species in a community. To estimate species evenness, we used the Pielou evenness index. To account for both abundance and evenness of the species present, we used the Shannon diversity index. We used the Bray-Curtis distance matrix to compare the similarity (beta diversity) among viral communities and generated non-metric multidimensional scaling (NMDS) plots. We were also interested in identifying the probably environmental sources of the viruses and their hosts. To do so, we first inferred the phage host from the viral name (e.g., Staphylococcus phage host would be *Staphylococcus* sp*.*, Lactococcus phage ul36 host would be *Lactococcus lactis*), and then, we looked up the sources of the bacteria via literature search (e.g., *Staphylococcus* sp. normally reside on the human skin, *Lactococcus lactis* are mainly isolated from plant material).

### Statistical analysis

We performed statistical calculations in the Rstudio. We constructed NMDS plots using the metaMDS function in the R vegan package. Bray-Curtis dissimilarity compares the community structures by taking into account the abundance distribution of viral species. We then used a variation of the BIO-ENV [74], routine dubbed BIO-BIO (http://menugget.blogspot.co.uk/2011/06/clarke-and-ainsworths-bioenv-and-bvstep.html), to identify the subset of viral species which best correlated to the overall biological pattern of the dissimilarity matrix. We overlaid the vectors of the best correlated biological variables on the NMDS plot. The length of the arrow is proportional to the correlation between the viral species and biological pattern of the dissimilarity matrix. Blue arrows indicate positive correlation (the highest viral relative abundance in that direction), and red arrows indicate negative correlation (the lowest viral relative abundance in that direction). We assessed variability in community structure by analysis of beta dispersion, which is based on the distances of each sample to its respective group centroid. Permutational Multivariate Analysis of Variance (PERMANOVA), aka Adonis, used Bray-Curtis dissimilarity measures to assess viral community compositional differences and its relationship to the different Seasons (999 permutations “vegan” package). We implanted the random forest classifier [75] in R using the “randomForest” library to identify viral species (present at least 10 times) that discriminate between the different seasons. Random forest is a supervised machine learning algorithm able to discriminate between two or more groups with high accuracy even in the presence of noise, high dimensional, and undersampled data, typical of biological problems [76]. Furthermore, the random forest can output the individual features (in our case viral hits) that contributed the most to the accuracy in discriminating between the groups (seasons).

## Additional file

Additional file 1: **Figure S1.** Heat map showing the abundance of the 500 most abundance DNA (A) and RNA (B) viruses. Both of our controls (unexposed filter and molecular biology grade water processed the same as the samples) showed extremely low raw BLASTN counts of DNA (mean = 100) and RNA (mean = 2) viruses. (PDF 637 kb)
