# Supplementary figures and images for: Seasonal dynamics of DNA and RNA viral bioaerosol communities in a daycare center
Source: Microbiome. 2019 Apr 1;7:53. doi: 10.1186/s40168-019-0672-z (PMC6444849; doi:10.1186/s40168-019-0672-z)

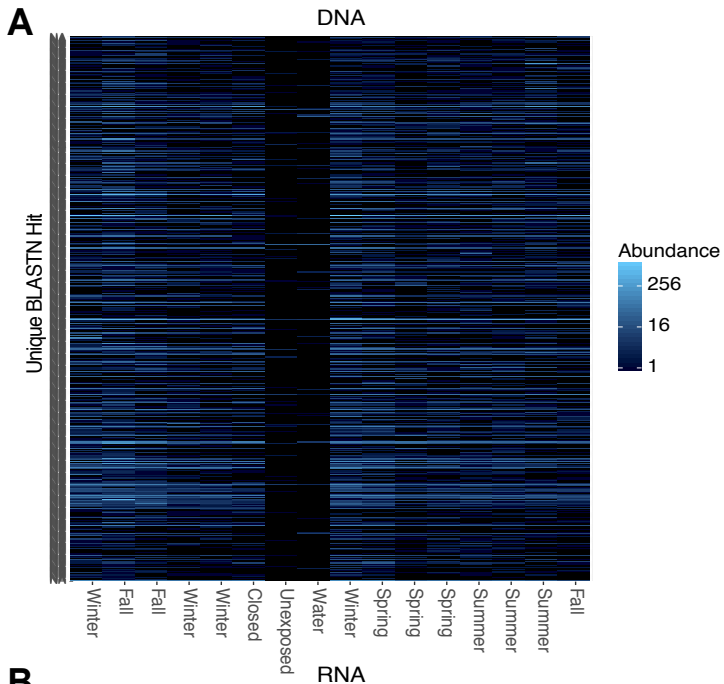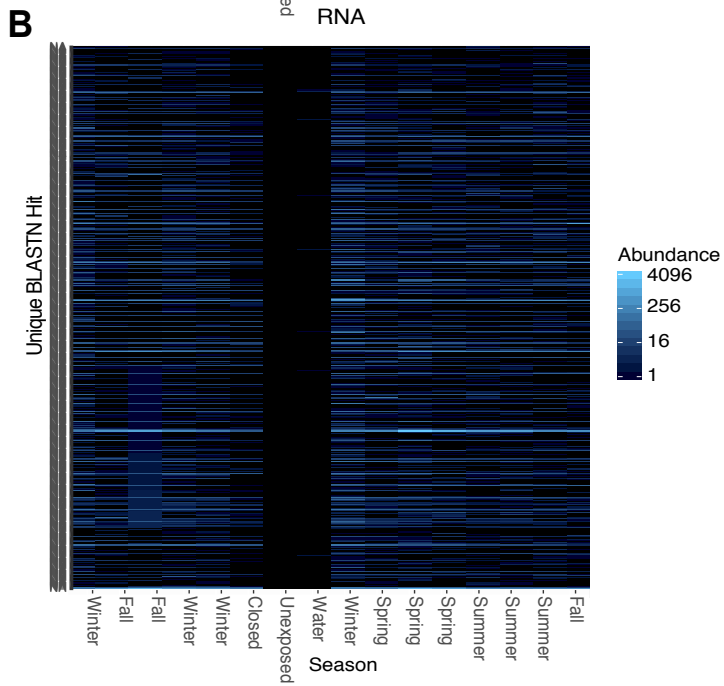

Supplement: Supplementary file 1 — Figure S1. Heat map showing the abundance of the 500 most abundance DNA (A) and RNA (B) viruses. Both of our controls (unexposed filter and molecular biology grade water processed the same as the samples) showed extremely low raw BLASTN counts of DNA (mean = 100) and RNA (mean = 2) viruses. (PDF 637 kb) [file 40168_2019_672_MOESM1_ESM.pdf]
